# The Urotensin II System and Carotid Atherosclerosis: A Role in Vascular Calcification

**DOI:** 10.3389/fphar.2016.00149

**Published:** 2016-06-07

**Authors:** Isabella Albanese, Stella S. Daskalopoulou, Bin Yu, Zhipeng You, Jacques Genest, Alawi Alsheikh-Ali, Adel G. Schwertani

**Affiliations:** ^1^Cardiology, McGill University Health CenterMontreal, QC, Canada; ^2^Cardiology, College of Medicine, Mohammed Bin Rashid UniversityDubai, UAE

**Keywords:** immunohistochemistry, calcium, URP, UT, UII, urotensin II, urotensin II-related peptide, smooth muscle cells

## Abstract

**Background and Aims:** The aims of the present study were to determine the expression of urotensin II (UII), urotensin-II related peptide (URP), and their receptor (UT) in stable and unstable carotid atherosclerosis, and determine the effects of UII on human aortic smooth muscle cell (SMCs) calcification.

**Methods and Results:** We examined UII, URP, and UT protein expression in 88 carotid endarterectomy specimens using immunohistochemistry. Expression of UII, URP, and UT was more evident in unstable compared to stable plaques (*P* < 0.05). Multivariate Spearman correlation analyses revealed significant positive correlations between UII, URP and UT overall staining and presence of calcification, severity of stenosis and inflammation (*P* < 0.05). Subjects undergoing carotid endarterectomy had significantly higher plasma UII levels, as assessed by ELISA, when compared with normolipidemic healthy control subjects (*P* < 0.05). Incubation of human aortic SMCs cultured in phosphate media with varying concentrations of UII resulted in a significant increase in calcium deposition and alkaline phosphatase activity. UII also significantly increased β-catenin translocation and expression of *ALPL, BMP2, ON*, and *SOX9* (*P* < 0.05). Incubation of cells with phosphate medium alone increased the expression of the pre-UT and mature UT (*P* < 0.01), and addition of UII had a synergistic effect on pre-UT protein expression (*P* < 0.001) compared to phosphate medium alone.

**Conclusions:** Upregulation of UII, URP, and UT in unstable carotid endarterectomy plaques and plasma, and the stimulatory effect of UII on vascular smooth muscle cell calcification suggest that the UII system may play a role in the pathogenesis of vascular calcification and stability of atherosclerosis.

## Introduction

Atherosclerosis is a leading cause of death in Western societies, and is a major contributor to several cardiovascular diseases such as myocardial infarction (MI) and stroke (Yusuf et al., 2002). Turbulent blood flow is a known endothelial cell activator, thus contributing to the formation of atherosclerotic plaques mainly in bifurcated blood vessels such as carotid arteries (Tsaousi et al., 2011). Endothelial dysfunction due to alterations in blood flow and/or vascular injury is considered the first step in the pathogenesis of atherosclerosis (Libby, 2012). The latter is characterized by the accumulation of lipids and fibrous lesions in large arteries (Lusis, 2000). Vascular calcification occurs in advanced atherosclerotic lesions, and is a marker of atherosclerosis associated with cardiovascular pathologies such as hypertension, congestive heart failure, hypertrophy, ischemia, myocardial infarction, and stroke (Johnson et al., 2006; Demer and Tintut, 2008). Long-term outcomes are worse in patients with calcified blood vessels (Shao et al., 2010).

Urotensin II (UII) is a potent vasoactive peptide, first isolated in the teleost fish *Gillichthys mirabilis* (Pearson et al., 1980), and subsequently identified in human and other species (Coulouarn et al., 1998; Ames et al., 1999; Elshourbagy et al., 2002). UII possess a wide range of vasoactive properties depending on the type of vessel and species (Itoh et al., 1988; Douglas et al., 2000; Affolter and Webb, 2001). Urotensin II related peptide (URP) is the endogenous and functional ligand for UII receptor (UT) in rats and mice, and its expression has been shown to be elevated in essential hypertension (Prosser et al., 2008). UII and UT have been associated with multiple cardiovascular pathologies such as congenital heart disease (Simpson et al., 2006), ischemic reperfusion injury (Prosser et al., 2008), atrial fibrillation (Zhang et al., 2009), and congestive heart failure (Douglas et al., 2002; Dschietzig et al., 2002; Dai et al., 2008). We were the first to demonstrate that atherosclerotic lesions of the human carotid arteries and aorta have increased expression of UII and UT compared to healthy vessels (Bousette et al., 2004). In recent years, significant evidence has begun to emerge about the role of the UT receptor system in atherosclerosis (Ross et al., 2010). We have demonstrated that both the UT antagonist SB657510A and UII gene deletion in atherosclerotic mice can lead to several beneficial effects, including reductions in serum cytokines, inflammatory adipokines, and adipogenesis, as well as improvement in hypertension and glucose tolerance (You et al., 2012). UT antagonist SB657510A also attenuates diabetes-associated plaque development (Watson et al., 2013). Despite the strong evidence implicating UII in atherosclerosis and associated features such as hyperlipidemia, there are currently no studies demonstrating a direct role for UII in vascular calcification.

In this study, we aimed to assess the tissue expression of UII, URP, and UT, and the plasma levels of UII in relation to carotid plaque stability, and presence of calcification, inflammation, and lipid. We also determined the effect of UII on human aortic SMCs differentiation, calcification, alkaline phosphatase (ALP) enzyme activity, and signaling pathway of genes associated with osteogenesis.

## Materials and methods

### Tissue collection

Eighty-eight carotid plaque specimens were freshly collected from patients who underwent carotid endarterectomy procedures between 2009 and 2011. Summary of clinical data for these patients is shown in Table 1. Venous blood samples were also collected from 55 of these patients (38 stable and 17 unstable) as well as 44 normolipidemic healthy control subjects into EDTA-containing tubes and were centrifuged and stored at −80°C for future analysis of plasma UII and biochemical analysis in batches (Table 2). The degree of carotid stenosis was assessed using 3D carotid ultrasound (Philips iu22). This study was approved by the McGill Ethics Institutional Research Board, and all patients provided written informed consent.

**Table 1:** **Clinical characteristics of the carotid endarterectomy specimens included in the immunohistochemical study**.

**Parameter**	**Stable (*N* = 57)**	**Unstable (*N* = 31)**	**Statistical difference**
Age (years)	68.74 ± 10.05	71.42 ± 7.61	NS
Sex (Number of females)	18 (31.6%)	6 (19.4%)	NS
BMI (kg/m^2^)	26.95 ± 3.97	26.65 ± 4.79	NS
TC (mmol/L)	3.68 ± 1.04	3.72 ± 1.08	NS
TG (mmol/L)	1.72 ± 0.96	1.70 ± 0.69	NS
HDL (mmol/L)	0.96 ± 0.27	0.93 ± 0.27	NS
LDL (mmol/L)	2.00 ± 0.95	2.01 ± 0.93	NS
Total Chol/HDL	4.04 ± 1.36	4.13 ± 1.06	NS
ApoB (g/L)	0.74 ± 0.24	0.77 ± 0.24	NS
ApoA1 (g/L)	1.23 ± 0.24	1.26 ± 0.30	NS
ApoB/ApoA1	0.62 ± 0.23	0.63 ± 0.20	NS
SBP (mmHg)	141.2 ± 16.39	139.7 ± 17.60	NS
DBP (mmHg)	70.07 ± 9.99	64.14 ± 9.98	NS
Stenosis (%)	84.5 ± 10.51	86.07 ± 8.94	NS
Diabetes (Number of patients)	19 (33.3%)	8 (25.8%)	NS

*BMI, body mass index; TC, total cholesterol; TG, triglycerides; HDL, high-density lipoprotein; LDL, low-density lipoprotein; ApoB, Apolipoprotein B; ApoA1, Apolipoprotein A1; SBP, systolic blood pressure; DBP, diastolic blood pressure; NS, not significant*.

**Table 2:** **Clinical characteristics of the carotid endarterectomy patients vs. control subjects included in the ELISA study**.

**Parameter**	**Carotid patients (*N* = 55)**	**Control (*N* = 44)**	**Statistical difference**
Age (years)	68.98 ± 9.30	41.41 ± 15.37	*P* < 0.0001
Sex (Number of females)	15 (27.3%)	20 (45.5%)	NS
BMI (kg/m^2^)	26.91 ± 4.01	23.02 ± 3.29	*P* < 0.0001
TC (mmol/L)	3.68 ± 1.01	4.90 ± 1.09	*P* < 0.0001
TG (mmol/L)	1.74 ± 0.89	1.46 ± 0.76	NS
HDL (mmol/L)	0.95 ± 0.26	1.23 ± 0.22	*P* < 0.0001
LDL (mmol/L)	1.97 ± 0.94	1.16 ± 0.38	*P* < 0.0001
Total Chol/HDL	4.08 ± 1.26	4.12 ± 1.27	NS
ApoB (g/L)	0.75 ± 0.24	1.03 ± 0.25	*P* < 0.0001
SBP (mmHg)	140.8 ± 18.3	117.2 ± 16.8	*P* < 0.0001
DBP (mmHg)	69.2 ± 10.0	72.3 ± 10.5	NS
Statins (Number of patients)	(38/55)	(4/44)	*P* < 0.0001

*BMI, body mass index; TC, total cholesterol; TG, triglycerides; HDL, high-density lipoprotein; LDL, low-density lipoprotein; ApoB, Apolipoprotein B; ApoA1, Apolipoprotein A1; SBP, systolic blood pressure; DBP, diastolic blood pressure; NS, not significant*.

### Immunohistochemistry

The paraffin-embedded carotid specimen blocks were cut into 4–5 μm sections using a microtome and placed on glass slides. The immunohistochemical technique used here had previously been described (Pearson et al., 1980). Five sections were examined per lesion (for each antiserum). A total of 6 images were analyzed per section.

The antibody used for the URP immunostaining studies was purchased from Phoenix Pharmaceuticals H-071-17, and does not cross react with human UII. The antibodies against UII and UT used have been previously described (Douglas et al., 2002; Bousette et al., 2006b). Additional sections were stained for α-smooth muscle actin (smooth muscle marker, Cedarlane CLT9000, 1/500) and Mac-2 (macrophage marker, Cedarlane CL8942AP, 1/2500).

Carotid plaques were semi-quantitatively assessed blindly by two independent vascular pathologists for calcification, fibrosis, inflammation, lipid, microvessels, hemorrhage, thrombus, cap infiltration with inflammatory cells and overall plaque instability, using well-established scales, including the widely used American Heart Association (AHA) plaque classification (Stary et al., 1995; Lovett et al., 2004). All plaques were classified as either AHA type V or type VI and lesions that were classified as type V were considered as stable and those classified as type VI, considered unstable. Immunostaining was assessed using Image J by two independent observers.

### Measurement of circulating UII levels

In all 55 subjects who underwent carotid endarterectomy, as well as in the normolipidemic healthy control subjects UII levels were measured using human urotensin II ELISA kit (PromoKine, catalog number: PK-EL-K101, Germany) in accordance with the manufacture's manual. The specificity of the ELISA kit had previously been described (Al Kindi et al., 2014).

### *In vitro* studies

#### *In vitro* calcification experiments

Human aortic SMCs were cultured in Medium-231 (ThermoFisher) including Smooth Muscle Cell Differentiation Supplement (ThermoFisher) in the presence of varying concentrations of UII (0, 10, 50, 100 nM), 100 nM UII + varying concentrations (0, 1, 10 μM) of UT antagonist SB657510A (kindly offered by GSK, USA), 0, 10, 50, 100 nM of URP, and 100 nM URP + 10 μM of UT antagonist SB657510A and 100 nM of URP for 2 weeks. Calcium content was measured using the Arsenazo III method. Cells were assayed in triplicates in 96 well plates.

Briefly, cells were washed three times with 2 ml PBS (Phosphate-Buffered Saline, Life Technologies) per well for 5 min each wash. Cells were then incubated with 250 μL of 0.6N HCl for 24 h at room temperature (RT) and Ca^2+^ was measured using colorometric Arsenazo III method. To the remaining cells, protein lysis buffer was added (100 μL of 0.1N NaOH with 0.1% SDS) for 20 min at RT. Cells were transferred to Eppendorf tubes and centrifuged for 5 min at 4°C at 15,000 rpm. The supernatants were removed and stored at −80°C for subsequent protein measurement, for the normalization of Ca^2+^measurements.

#### *In vitro* ALP assay

SMCs were incubated for 7 days in the following conditions: DMEM alone. Forty-eight hours after incubation under the different media conditions, ALP activity in human aortic smooth muscle cell media was assessed using Alkaline Phosphatase Activity Colorimetric Assay Kit (BioVision) according to manufacturer protocols. Eighty microliters of media was added to each well. Eighty microliters of fresh media were also added to separate wells to use as a sample background control. Twenty microliters of Stop Solution was added to the background control. Then, 50 μL of 5 mM p-nitrophenyl phosphate (pNPP) was added to each well and reaction was incubated for 1 h at 25°C, protected from light. Twenty microliters of Stop Solution was added to each sample and standard well to stop the reactions. O.D. was measured at 405 nm in micro plate reader. Using standard curve, ALP activity in the media of each sample was calculated and is expressed in Glycine Units. Glycine Units are defined as the amount of enzyme causing hydrolysis of 1 μmol of pNPP per minute at pH 9.6 and 25°C.

#### Effect of phosphate medium on UT protein expression in SMCs

SMCs were incubated in either DMEM, DMEM+UII (100 nM), Pi-DMEM (phosphate: 2.6 mmol/l), or Pi-DMEM+UII (phosphate: 2.6 mmol/l, UII: 100 nM) for 7 days. The medium was changed every 2–3 days. After two washes in PBS buffer, the cells were placed in lysis buffer for 15 min on ice. The solutions were transferred into eppendorf tubes and centrifuged at 8000 rpm for 5 min. UT expression was detected with Western blot (primary antibody dilution 1:1000, and secondary antibody dilution 1:5000). UT antibody used was as previously described (Bousette et al., 2006a).

*GAPDH* was as internal standard. Image J was used to quantity UT expression.

#### Effect of UII on SMC proliferation

Vascular SMCs were incubated with phosphate medium with or without 10 nM UII for 4 and 7 days. Cells were then fixed in 4% paraformaldehyde and stained with antiserum to α-SMC actin using the avidin-biotin-peroxidase method described above. The numbers of α-SMC actin-positive cells were quantified using Image J.

#### RT-PCR analyses

Vascular SMCs were treated with or without 100 nM human UII peptide (Sigma) for 24 h in osteogenic medium (OSM). Cells in 24-well plate were washed with cold PBS, then total RNA were isolated by using RNeasy mini kit(Qiagen). One microgram RNA was used for the first strand cDNA synthesis using iScript Select cDNA synthesis kit (Bio-Rad), and qPCR performed using Advanced qPCR Master mix HI-ROX(Wisent) on StepOneTM Plus (Applied Biosystems) for various osteogenic mediators. Gene expression level was calculated against a control standard curve generated by housekeeping gene: *GAPDH*, relative expression level is presented as relative ratio normalized against sample *GAPDH* level. DMEM medium (DM): Gibco DMEM with high glucose containing 10% FBS, 1% Penicillin, 1% Streptomycin; Osteogenic medium: full DMEM medium with calcification reagent, 2 mM phosphate buffer and 0.2 mM ascorbic acid. *N* = 4 per experiment.

To assess UII, URP, and UT mRNA expression in carotid plaques, total RNAs (total 20 IHC matched samples, stable cases = 10, unstable cases = 10) were extracted from surgically removed carotid plaques using TRIzol(ThermoFisher) and RNeasy mini kit(Qiagen). First strain cDNAs were synthesized using 1 μg total RNA with iScript Select cDNA synthesis kit (Bio-Rad), and qPCR performed using Advanced qPCR Master mix HI-ROX(Wisent) on StepOneTM Plus (Applied Biosystems). GAPDH was used as internal control, relative gene expression was calculated using GeneStudy software (Bio-Rad).

#### Effect of UII on β-catenin nuclear translocation

Vascular SMCs were seeded in duplicates of 100 mm petri dishes and were pre-incubated in osteogenic medium for 4 days, then UII (100 nM) or URP (100 nM) was added into medium for 20 h. Nuclei were extracted with Nuclear Extract Kit (Active Motif, CA USA, cat#40010). Nuclear protein was loaded on SDS-PAGE, and β-catenin was detected by Western blot using a polyclonal antiserum (HPA029159, Sigma). The membranes were probed with antiserum to TFIIB as an internal standard (Santa Cruz).

### Statistical analysis

Multivariate Spearman analysis was used to assess correlations between UII, URP and UT immunohistochemical stainings with the histologic features of carotid atherosclerosis (degree of stenosis, extent of calcification, fibrosis, inflammation, lipid score, microvessels, hemorrhage, thrombus, and cap instability) and clinical parameters [age, sex, body mass index (BMI), total cholesterol, triglycerides, HDL, LDL, Total Chol/HDL, ApoB, ApoA1, ApoB/ApoA1, stenosis, and blood pressure]. One-way ANOVA was used to assess UII, URP, and UT immunostaining in stable compared to unstable carotid plaques, plasma UII measurements in stable and unstable carotid endarterectomy patients as well as when comparing plasma UII values to controls, and the significance of the differences in SMC calcification and ALP activity for different stimulation conditions. For the clinical comparison of patient populations, unpaired *t*-test was performed to determine the statistical significance of continuous variables between the groups. Fisher's exact test was performed to determine the statistical significance of the categorical variables (sex, hypertension, and diabetes) between the two groups. Statistical analyses were performed using GraphPad Prism software version 6.0d (GraphPad Software Inc., La Jolla, CA, USA). *P* < 0.05 was considered significant.

## Results

### UII immunohistochemistry

**Figure 1:**
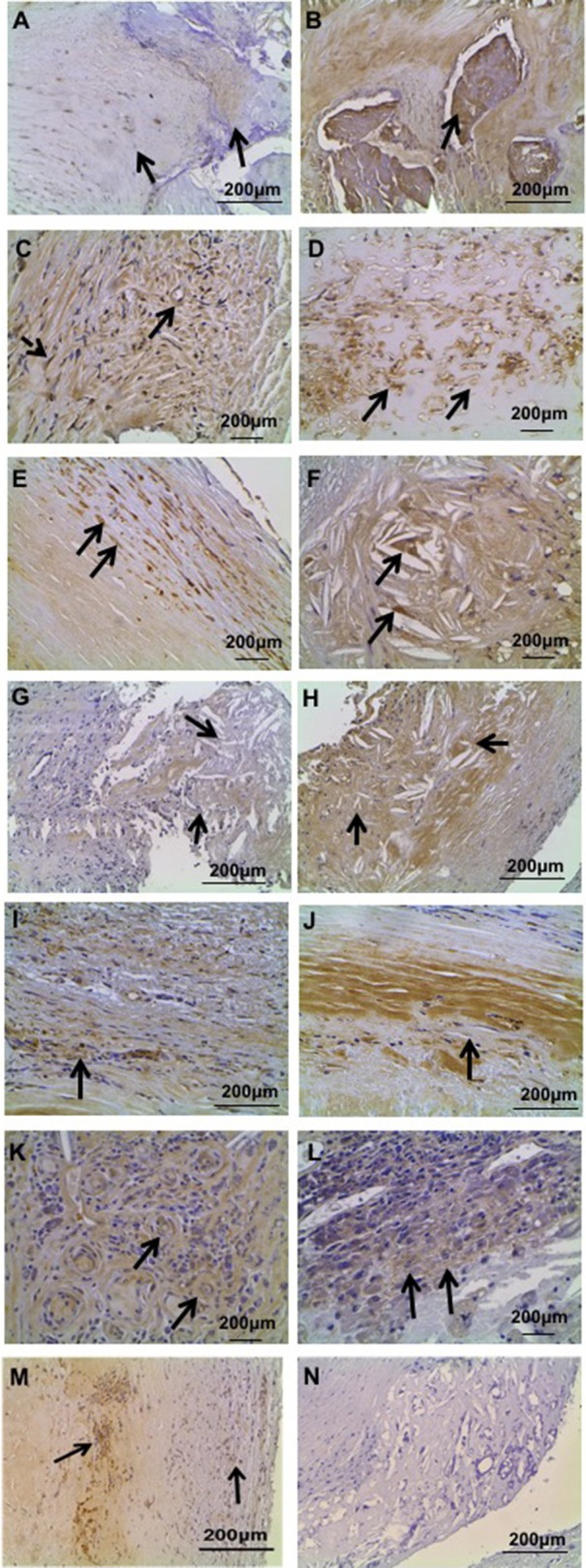
**Representative immunohistochemical localization of UII, UT, and URP in human carotid plaque specimens. (A)** UII immunoreactivity in stable carotid plaque: low intensity UII immunostaining in calcification (right arrow) and higher intensity immunostaining in myointimal cells (left arrow). **(B)** Strong UII immunoreactivity in calcified lesions of unstable carotid plaque. **(C)** UII staining in microvessels (bold arrow) and myointimal cells (dashed arrow) in stable carotid plaque. **(D)** Abundant UII staining in microvessels of unstable plaque. **(E)** UII staining in media SMCs of unstable plaque. **(F)** UII staining in foam cells of unstable plaque. **(G)** URP immunoreactivity in lipid core and surrounding cholesterol clefts (arrows) in stable carotid plaque. **(H)** Strong URP immunoreactivity in lipid core of unstable carotid plaque. **(I)** URP immunoreactivity in fibrosis of stable plaque. **(J)** Elevated URP immunoreactivity in fibrosis of unstable plaque. **(K)** URP staining in microvessels of unstable plaque. **(L)** URP staining in inflammatory cells of unstable plaque. **(M)** Abundant UT immunoreactivity in myointimal cells (right arrow) and calcium deposition (left arrow). **(N)** Negative control section of lipid and myointimal cells immunostained with normal serum showing no immunoreactivity.

UII immunostaining was highest in myointimal cells, followed by media SMCs and foam cells (Figures 1A,E,F). Importantly, UII immunostaining intensity was significantly elevated in activated myointimal cells compared to myointimal cells that were not classified as activated (*P* < 0.0001). Semi-quantitative analyses of UII immunostaining intensity showed that UII immunoreactivity was significantly higher in osteoblast, medial smooth muscle cells and microvessels in unstable compared to stable carotid plaques (*P* < 0.05; Figures 1A–D; Supplemental Figure 1). Multivariate Spearman correlation analysis revealed significant correlations between overall UII immunoreactivity and diastolic blood pressure (*r* = 0.2410; *P* < 0.05), calcification (*r* = 0.268; *P* < 0.05), remodeling (*r* = 0.339, *P* < 0.01), inflammation (*r* = 0.5077; *P* < 0.0001), lipid (*r* = 0.353; *P* < 0.001), URP immunoreactivity (*r* = 0.671; *P* < 0.0001), and UT immunoreactivity (*r* = 0.440; *P* < 0.0001).

### URP immunohistochemistry

URP immunostaining was evident in endothelial, myointimal, medial SMCs, and inflammatory cells. URP was also present in microvascular endothelial cells, foam cells and osteoblasts (Figures 1G–L). Semi-quantitative analyses of URP immunostaining intensity showed that URP immunoreactivity was significantly elevated in endothelial and foam cells of unstable compared to stable plaques (*P* < 0.05; Figures 1G–L; Supplemental Figure 1). Multivariate Spearman correlation analysis revealed significant correlations between overall URP immunoreactivity and calcification (*r* = 0.261; *P* < 0.0127), fibrosis (*r* = 0.276; *P* < 0.01), remodeling (*r* = 0.2793; *P* < 0.05), inflammation (*r* = 0.489; *P* < 0.0001), lipid (*r* = 0.304; *P* < 0.01), and UT immunoreactivity (*r* = 0.4; *P* < 0.001).

### UT immunohistochemistry

UT cellular expression showed strongest immunostaining in myointimal cells followed by medial SMCs (Figure 1M). Semi-quantitative analyses of UT immunostaining showed no significant differences in stable compared to unstable carotid plaques (Supplemental Figure 1). Multivariate Spearman correlation analysis revealed significant correlations between UT immunoreactivity and BMI (*r* = −0.2431; *P* < 0.05), carotid stenosis (*r* = 0.2669; *P* < 0.05), calcification (*r* = 0.381; *P* < 0.001), fibrosis (*r* = 0.2175, *P* < 0.05), remodeling (*r* = 0.297; *P* < 0.01), and inflammation (*r* = 0.292; *P* < 0.01).

Negative control sections immunostained with the non-immune sera or preabsorped with the respective antigens did not show immunoreaction (Figure 1N).

### RT-PCR

UII, URP, and UT mRNA expression was also assessed in stable and unstable carotid plaques however there was no significant difference between the two groups in any of the three molecules (Supplemental Figure 2).

### Plasma UII levels

The mean age of the carotid endarterectomy patients included in the measurement of plasma UII was 69.0 ± 9.3 years and was significantly higher than the control group (41.4 ± 15.4 years; *P* < 0.0001). Plasma UII levels were still significantly elevated in carotid endarterectomy patients compared to an age-matched control subgroup (*n* = 10; *P* < 0.0001). BMI, LDL, systolic blood pressure, and statin use were significantly higher in the carotid endarterectomy group; while total cholesterol, HDL, and ApoB were significantly lower when compared with that of the control group (see Table 2 in Materials and Methods). The mean (±SEM) plasma level of UII in carotid endarterectomy patients was significantly elevated (1194.0 ± 83.8 pg/ml) compared to the control group (252.7 ± 13.9 pg/ml; *P* < 0.0001). Plasma UII levels were still significantly elevated in carotid endarterectomy patients compared to an age-matched control subgroup (293.1 ± 36.92 pg/ml, *n* = 10; *P* < 0.0001). Correlation studies revealed significant correlations between plasma UII and age (*r* = 0.597, *P* < 0.0001), BMI (*r* = 0.453, *P* < 0.0001), systolic blood pressure (*r* = 0.5130, *P* < 0.0001), total cholesterol (*r* = −0.35; *P* < 0.001), triglycerides (*r* = 0.274, *P* < 0.01), HDL (*r* = −0.439, *P* < 0.0001), and LDL (*r* = 0.558, *P* < 0.0001).

### *In vitro* data

**Figure 2:**
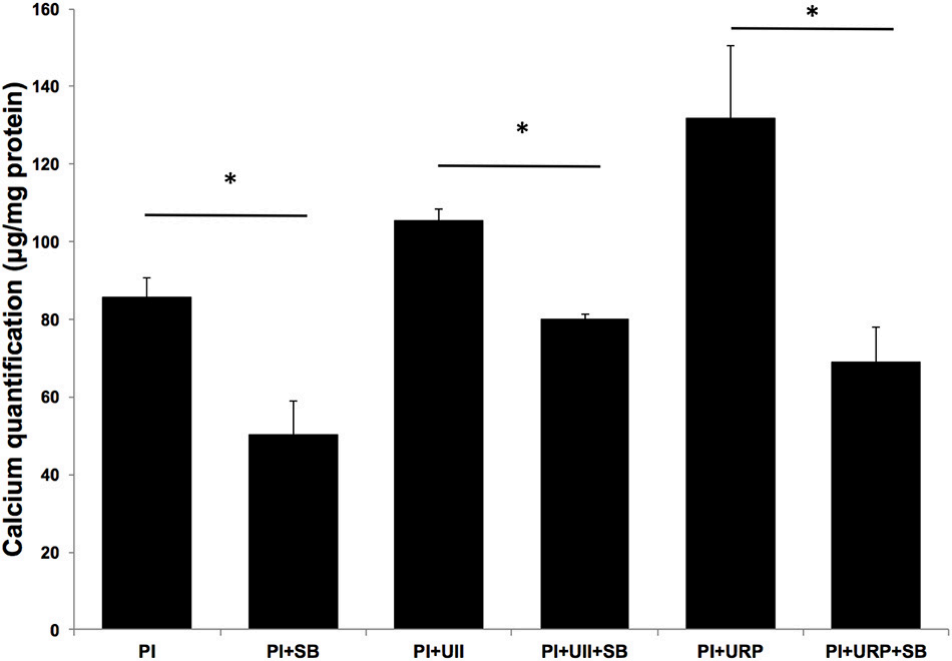
**UT Antagonist SB657510A (SB) inhibits UII and URP induced SMC calcification**. SMCs were incubated in phosphate medium (PI) for 14 days with 100 nM UII or 100 nM URP, in the presence and absence of UT antagonist (SB) at concentration 10 μM. UII and URP stimulatory effect on SMC calcification was abolished in the presence of SB657510A. ^*^P < 0.05 (*n* = 4 per experiment).

Vascular SMCs were incubated in phosphate medium for 2 weeks with varying concentrations of urotensin II (0, 10, 50, 100 nM). SMC calcification was assessed as the ratio of calcium (μg)/total protein (mg). While supplementation with UII had no significant effect on calcification in normal media, UII appears to be potentiating the effects of phosphate medium on SMC calcification in a dose-dependent manner. Phosphate medium + 100 nM UII had significantly elevated calcium deposition compared to phosphate medium alone (*P* = 0.0167, Figure 2). URP at 100 nM concentration showed the strongest stimulation of SMC calcification (*P* = 0.0376, Figure 2).

UII and URP stimulatory effect on SMC calcification were significantly reduced in the presence of SB657510A at 10 μM concentration (*P* = 0.0051 and *P* = 0.0191; respectively, Figure 2). This demonstrates that UII and URP effects on SMC calcification are mediated by signaling through UT.

**Figure 3:**
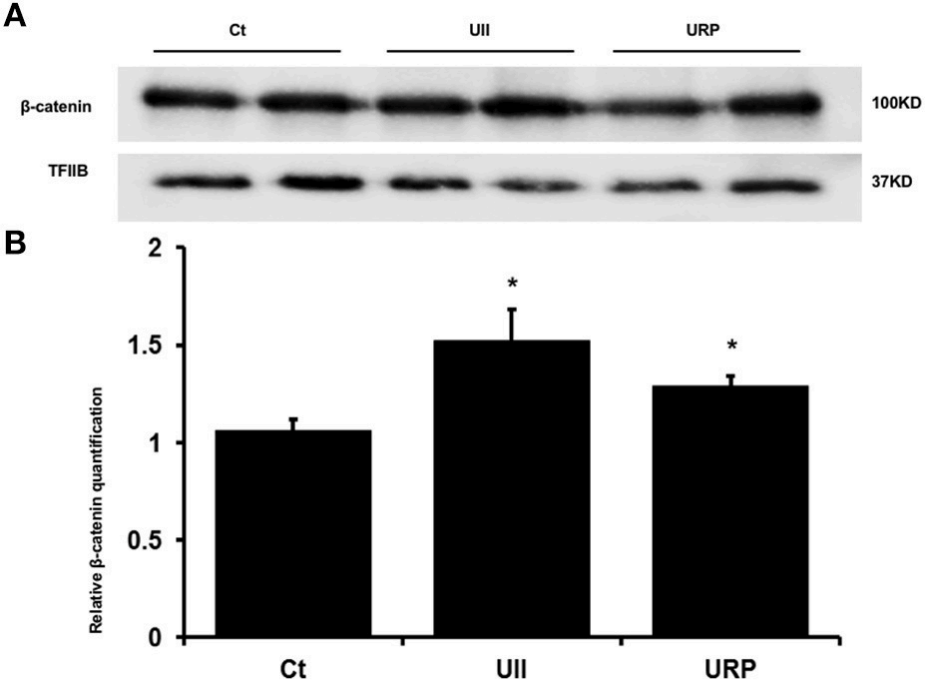
**UII and URP induce SMC nuclear translocation of β-catenin**. SMCs were incubated in phosphate medium(PI) in the presence and absence of 100 nM UII or 100 nM URP. Incubation of SMCs with UII and URP each significantly induced β-catenin nuclear translocation. Nuclear β-catenin protein expression was normalized to the expression of TFIIB (Transcription Factor IIB). ^*^P < 0.05 (*n* = 6 per experiment). **(A)** Western blot visualization. **(B)** Relative β-catenin quantification normalized to the expression of TFIIB (Transcription Factor IIB).

There was a significantly reduced number of α-smooth muscle actin (α-SMA) positive cells 4 days after incubation with phosphate medium containing 100 mM UII compared to phosphate medium alone (data not shown; *P* < 0.01). At 7 days there was no difference between phosphate medium with or without UII. Moreover, incubation of SMCs with UII as well as URP significantly induced β-catenin nuclear translocation (*P* < 0.05) (Figure 3).

**Figure 4:**
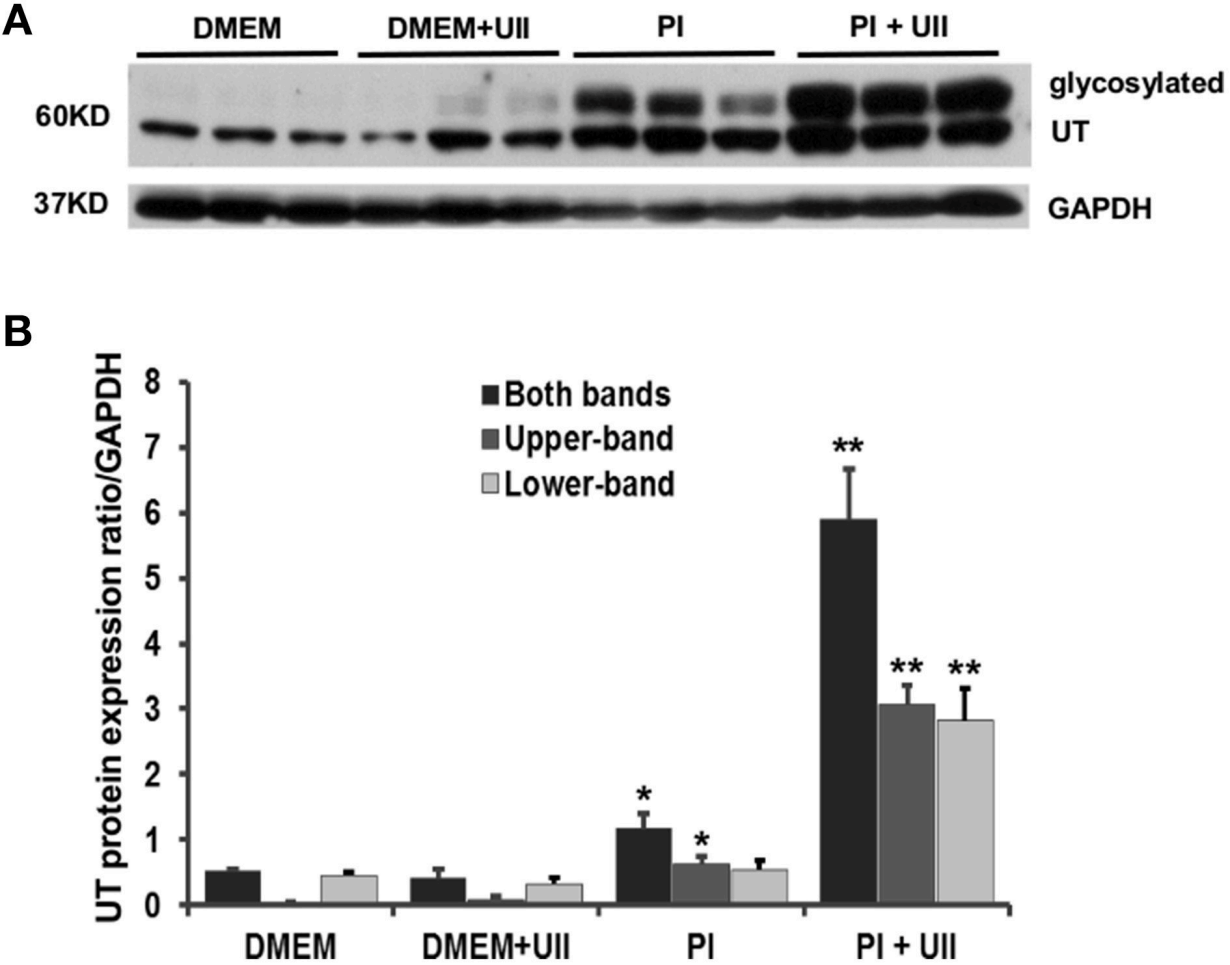
**Phosphate medium induces increased protein expression of UT**. SMCs were incubated under one of the following conditions: DMEM alone, DMEM + 100 nM UII, phosphate medium (Pi) alone or phosphate medium (Pi) + 100 nM UII. Two different degree of glycosylated UT (Upper-band and Lower-band) were detected around 60KD in SMCs. Phosphate medium significantly increases the total UT and Upper-band compared to DMEM and DMEM + UII conditions, ^*^P < 0.01. UII stimulation significantly increased UT expression in Pi medium compared to Pi medium alone ^**^P < 0.001. *N* = 4 for each condition. **(A)** Western blot visualization. **(B)** Relative UT quantification normalized to the expression of GAPDH.

Assessment of UT protein expression in SMCs following incubation with phosphate medium revealed a significant increase in protein expression of UT, both mature and immature forms (Figure 4). UII stimulation potentiates the phosphate medium-induced increase in pre-UT as the phosphate medium + UII stimulated cells had significantly elevated expression of pre-UT compared to phosphate medium alone. However, this was not the case with the mature form of UT, where both phosphate alone and phosphate + UII had significantly elevated mature UT protein expression compared to control conditions.

**Figure 5:**
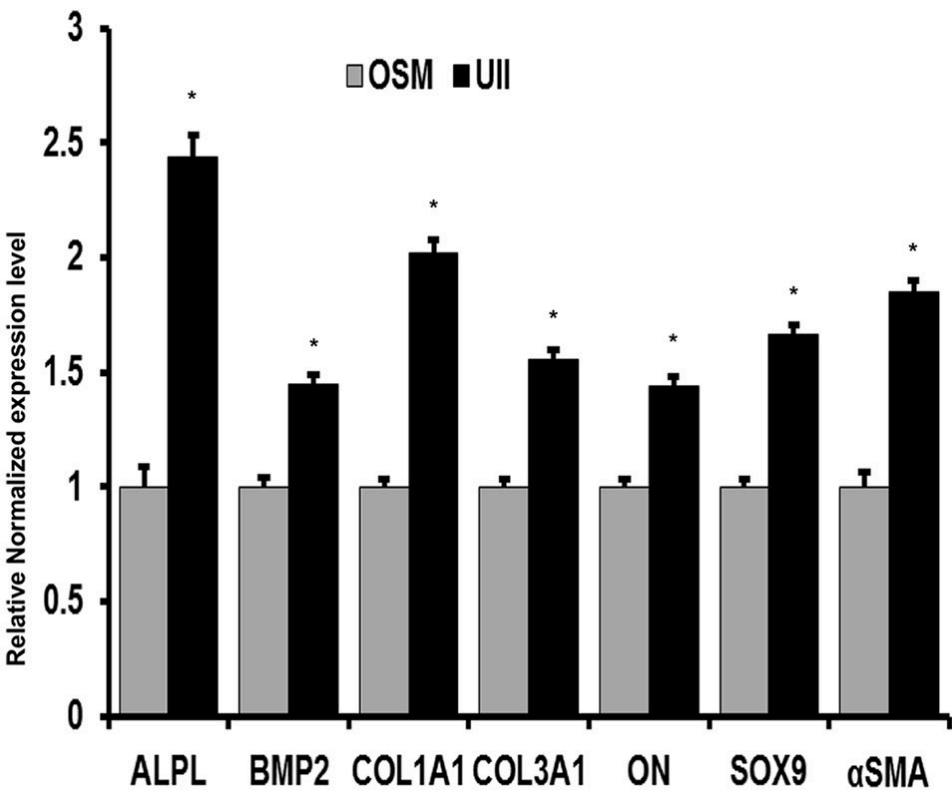
**UII stimulation of SMCs induces expression of osteogenic markers**. SMCs in phosphate medium (PI) were incubated with 200 nM UII for 24 h and expression of ALPL, BMP2, osteonectin, SOX9, collagen 1A1, collagen 3A1, and α-smooth muscle cell actin were assessed by Real time qPCR. Relative gene expression level was calculated against GAPDH level, and data is presented as fold change of relative interested gene expression in UII treated (UII). ^*^P < 0.01 (*n* = 4 per experiment).

Incubation of SMCs with phosphate medium significantly increased the expression of various osteogenic mediators. Addition of 100 nM UII for 24 h significantly augmented the expression of *ALPL, BMP2*, osteonectin (ON), *SOX9*, collagen 1A1, collagen 3A1, and α-SMC actin (*P* < 0.01) (Figure 5). Expression of *Msx2* and *Runx2* were not significantly altered.

## Discussion

There is an abundance of evidence showing that carotid atherosclerosis is associated with increased risk of stroke and mortality, particularly in elderly patients (Stary et al., 1995; Störk et al., 2004). There is currently a great need for clinically-relevant serum and tissue biomarkers of carotid atherosclerosis, especially those that can distinguish between stable and unstable carotid plaques. This will contribute to a greater understanding of the pathogenesis of this process and ideally, be a useful tool in the selection of patients for surgical intervention. In this study, we aimed at determining the serum level of UII and tissue expression of UII, URP, and UT in carotid plaque specimens in relation to their stability and to determine if UII plays a role in vascular SMC calcification.

Circulating levels of UII were higher in carotid endarterectomy compared with normal control subjects. We found significant differences in UII and URP immunostaining in unstable compared to stable plaques. Our *in vitro* studies revealed that UII and URP had a significant dose-dependent effect on SMC calcification in the presence of phosphate media and that this effect was abolished in the presence of UT antagonist SB657510A. UII stimulation also increased ALP activity and a number of osteogenic mediators in SMCs in the presence of phosphate media. Together, these results suggest that the UII stimulates the osteogenic differentiation of SMCs, and suggest a role for the UII system in the pathology of carotid plaques.

To our knowledge, there is currently little evidence for the role of URP in any features of atherosclerosis, and this is the first study profiling its tissue expression in carotid atherosclerosis. The major difference in URP expression in stable compared to unstable plaques are that unstable plaques had significantly higher intensity in endothelial and foam cells. URP immunoreactivity was also very strong in osteoblasts, macrophages and lymphocytes and activated myointimal cells, however this was not significantly different in stable compared to unstable plaques. There is currently very little work on differential expression of UII and URP. Studies in mice have shown similar UII and URP expression in the nervous system, skeletal muscle, testes, vagina, and gall bladder, and differential expression in the seminal vesicles, heart, colon, and thymus (Dubessy et al., 2008). Another study of spontaneously hypertensive rats found elevated URP mRNA expression, suggesting a potential role for URP in spontaneous hypertension (Forty and Ashton, 2013). The differential cellular expression of URP in unstable vs. stable carotid atherosclerosis suggests that this may occur as either cause or effect in atherosclerotic disease and warrants further investigation.

We have previously demonstrated that UII and UT immunostaining is significantly elevated in atherosclerotic human carotid arteries compared to control, citing immunoreactivity in endothelial, smooth muscle, and inflammatory cells (Bousette et al., 2004). In this study, we analyzed tissue expression relative to the stability of carotid plaques and found significantly elevated intensity in osteoblasts and medial SMCs in unstable compared to stable plaques. UII and UT immunoreactivities were also very strong in inflammatory cells of both stable and unstable plaques but since unstable plaques had significantly more inflammation, they had greater overall UII and UT immunostaining in these cells. Given that unstable plaques are associated with increased inflammation and release of cytokines, it is reasonable to assume that the increase in UII and UT is driven by inflammatory cytokines such as IL-6, previously shown to induce the expression of these molecules (Zhou et al., 2012). Further investigations using animal models of unstable plaques are needed to determine the exact role of UII and UT in atherosclerotic plaque stability.

ELISA measurements of plasma UII levels in the carotid endarterectomy patients revealed elevated plasma UII compared to controls; however, there was no significant difference between plasma UII levels in stable and unstable patients. We found significant positive correlations between plasma UII and age, BMI, triglycerides, and systolic blood pressure, and significant negative correlations with HDL and total cholesterol. Our findings are consistent with previous studies in hypertensive carotid endarterectomy demonstrating increased UII and association with BMI, age and systolic blood pressure (Cheung et al., 2004; Chen et al., 2009). The inverse correlation between serum UII and total cholesterol and HDL can be attributed to statin use in patients with carotid endarterectomy, and further supported by our previous findings in experimental animals (You et al., 2012, 2014). Our findings that plasma UII is elevated in carotid atherosclerosis but not significantly different in stable compared to unstable patients demonstrate the importance of looking at tissue expression of UII and associated proteins to get better insight into their roles in the pathogenesis of carotid atherosclerosis.

This is the first study showing direct effects of UII and URP on SMC calcification and osteogenic differentiation. Vascular calcification occurs in very advanced atherosclerotic lesions and is a marker for atherosclerosis associated with several cardiovascular pathologies including hypertension, congestive heart failure, cardiac hypertrophy and ischemia and increased risk of myocardial infarction and stroke (Johnson et al., 2006; Demer and Tintut, 2008). It has been previously demonstrated that translocation of β-catenin and expression of skeletal morphogen BMP2 mediate the osteogenic differentiation of activated SMCs and this plays a crucial role in atherosclerotic calcification (Boström et al., 1993; Montes de Oca et al., 2014). Additionally, other osteogenic markers, osteonectin and SOX9, have also been localized to atherosclerotic plaques suggesting roles for them in the pathological calcification (Aigner et al., 2008; Farrokhi et al., 2014). Here, we demonstrate that UII and URP, in the presence of phosphate medium, stimulate calcium deposition in SMCs. Furthermore, UII increased ALP activity, β-catenin translocation, and increased expression of *ALPL, BMP2, ON*, and *SOX9*, thus indicating osteogenic differentiation. Importantly, we found that phosphate medium alone or with UII increased UT protein expression, thus providing support for an important role for the UII system in vascular calcification.

Limitations of this study: Increasing the number of patients included in the UII ELISA study may reveal differences in serum UII in patients with stable compared to unstable plaques. Additionally, further investigations should include the correlation of urotensin II levels with both tissue and serum levels of mediators of SMC osteogenic differentiation. Finally, the fact that our study only addresses the relationship between UII, URP and UT with atherosclerotic intimal calcification without any investigation of medial vascular calcification that is commonly seen in ESRD, diabetes, and elderly populations.

In summary, the present study demonstrates for the first time two important findings; first, local expression of the UII system in unstable carotid plaques is significantly higher than in stable plaques, and second, *in vitro* UII in the presence of phosphate medium induces ALP activity, β-catenin translocation, osteogenic transdifferentiation and ectopic calcium deposition in SMCs. A role for UII in inflammation (Liang et al., 2013), angiogenesis (Spinazzi et al., 2006), and increased release of metalloproteases (Zhou et al., 2012) has previously been established. Interestingly, atherosclerotic plaque instability is associated with increased inflammation (Shah, 2014), angiogenesis (Lam et al., 2013), and extracellular matrix protein degradation (Liu et al., 2014). Therefore, the present findings suggest that initially UII contribute to vascular calcification, however, sustained production and release of the peptide could contribute to plaque instability.

## Author contributions

All authors contribute to design, analysis, and writing of this work. IA, BY, ZY performed experiments.

### Conflict of interest statement

The authors declare that the research was conducted in the absence of any commercial or financial relationships that could be construed as a potential conflict of interest.

## Abbreviations

SMCs: Smooth muscle cells.
