# Supplementary figures and images for: The Urotensin II System and Carotid Atherosclerosis: A Role in Vascular Calcification
Source: Front Pharmacol. 2016 Jun 7;7:149. doi: 10.3389/fphar.2016.00149 (PMC4894881; doi:10.3389/fphar.2016.00149)

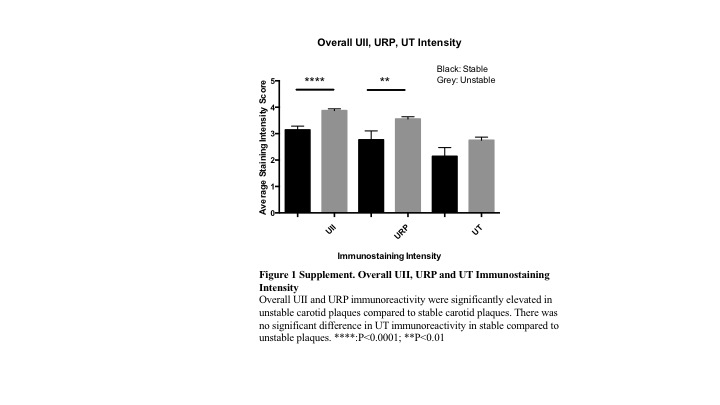

Supplement: Supplementary file 1 [file Image1.JPEG]

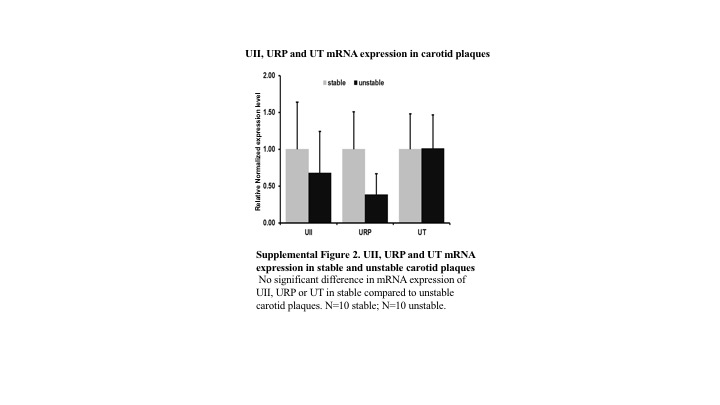

Supplement: Supplementary file 2 [file Image2.JPEG]
